# Supplementary material for: Household triclosan and triclocarban effects on the infant and maternal microbiome
Source: EMBO Mol Med. 2017 Oct 13;9(12):1732–41. doi: 10.15252/emmm.201707882 (PMC5709730; doi:10.15252/emmm.201707882)
Supplement: Supplementary file 1 — Appendix [file EMMM-9-1732-s001.pdf]

Appendix

Household triclosan and triclocarban effects on the infant and maternal microbiome

Jessica V. Ribado[1], Catherine Ley[2], Thomas D. Haggerty[2], Ekaterina Tkachenko[3], Ami S. Bhatt[1,3*], Julie Parsonnet[2,4*]

[1]Department of Genetics, Stanford University, Stanford, CA, USA.
[2]Division of Infectious Diseases and Geographic Medicine, Department of Medicine, Stanford University, Stanford, CA, USA.
[3]Division of Hematology, Department of Medicine, Stanford University, Stanford, CA, USA.
[4]Division of Epidemiology, Department of Health Research and Policy, Stanford University, Stanford, CA, USA.

*Correspondence to:
Ami S. Bhatt, M.D., Ph.D.
asbhatt@stanford.edu
Julie Parsonnet, M.D.
parsonnt@stanford.edu

Table of Contents

Appendix Figure S1…………………………………………………………………………….. 2
Appendix Figure S2…………………………………………………………………………….. 3
Appendix Table S1……………………………………………………………………………... 4
Appendix Table S2……………………………………………………………………………... 5

Appendix Figure S1: Distribution of sample collection relative to infant birth. Households for each visit time point are sampled approximately within one month, and are balanced between treatments in the study.

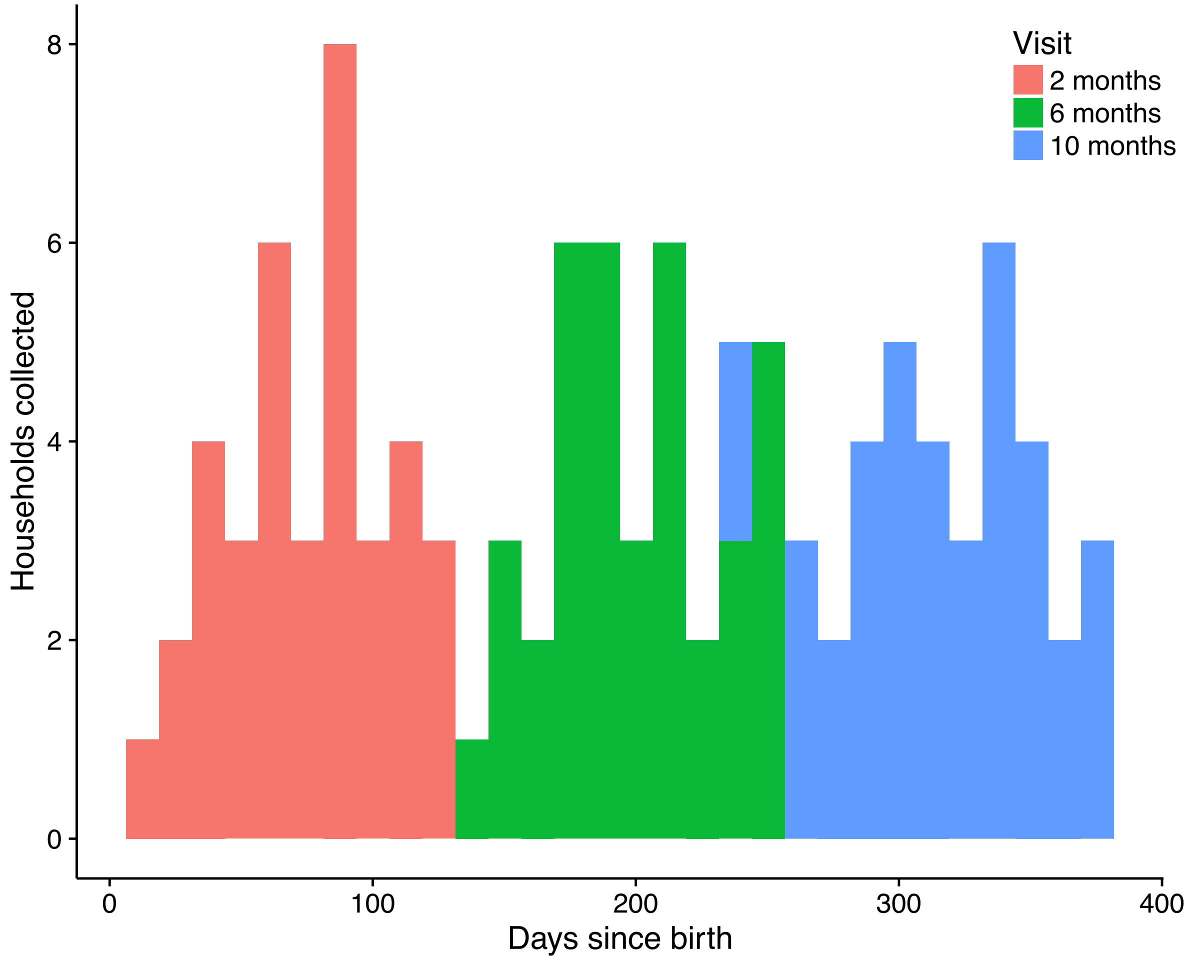

**Visit (Months)**	Treatment	Mean	Median	Minimum	Maximum	**Standard Deviation**
2	nTC	72.05	74	27	122	26.35
	TC	76.25	88	14	124	33.88
6	nTC	202.00	199	146	255	33.31
	TC	196.39	192	135	256	33.06
10	nTC	313.79	319	241	377	37.80
	TC	320.21	325	259	375	36.07

Appendix figure S2: DADA2 and BaseSpace RDP algorithms comparably capture microbiome variance. Principal coordinates analyses of Bray-Curtis dissimilarity of “core” or “developmental” taxa show that gut communities cluster by mothers and infants. **(A)** DADA2 and **(B)** BaseSpace RDP are comparable in first and second axis variance.

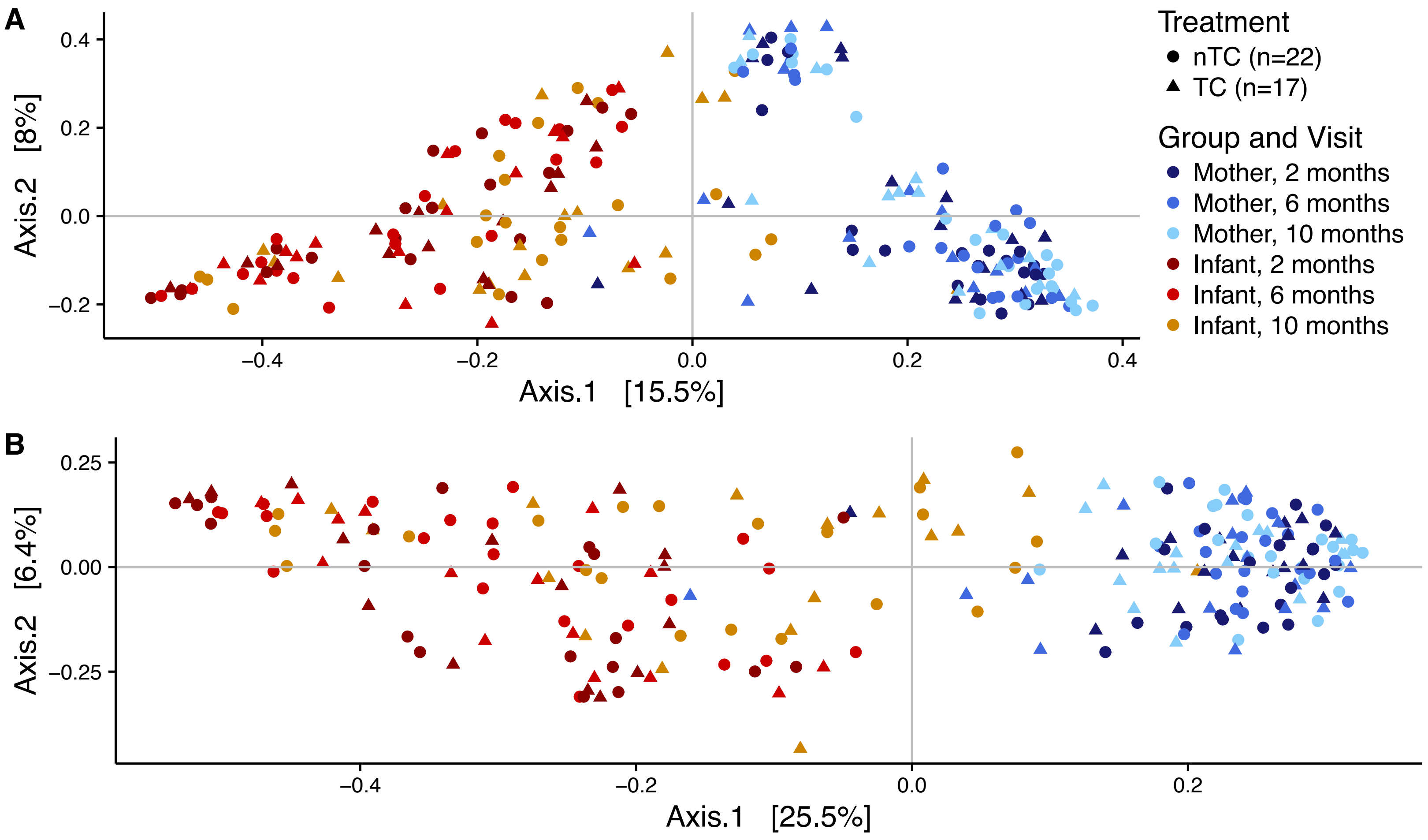

Appendix Table S1: Differentially abundant taxa for mothers and infants in the lower v. upper tertiles of urinary triclosan levels at 6 months (FDR adjusted p-value < 0.1). Analyses included 24 mothers (lower < 81.41 pg/microL, Upper > 597.46 pg/microL) and 20 infants (lower < 10.20 pg/microL, upper > 34.73 pg/microL).

Group	Log_2 fold change (High/Low)	Adjusted p-value	Phylum	Class	Order	Family	Genus	Species
Mother	-5.916	0.012	Bacteroidetes	Bacteroidia	Bacteroidales	Bacteroidaceae	Bacteroides	massiliensis
	-4.107	0.094	Bacteroidetes	Bacteroidia	Bacteroidales	Prevotellaceae	Prevotella	stercorea
	-3.769	0.068	Bacteroidetes	Bacteroidia	Bacteroidales	Bacteroidaceae	Bacteroides	stercoris
	-2.866	0.068	Bacteroidetes	Bacteroidia	Bacteroidales	Bacteroidaceae	Bacteroides	dorei
	-2.177	0.068	Bacteroidetes	Bacteroidia	Bacteroidales	Bacteroidaceae	Bacteroides	sartorii
	-1.669	0.068	Firmicutes	Clostridia	Clostridiales	Lachnospiraceae	Lachnospira	pectinoschiza
	-1.371	0.068	Firmicutes	Clostridia	Clostridiales	Clostridiaceae	Clostridium	
	2.864	0.012	Bacteroidetes	Bacteroidia	Bacteroidales	Bacteroidaceae	Bacteroides	denticanum
	3.628	0.094	Bacteroidetes	Sphingobacteriia	Sphingobacteriales	Sphingobacteriaceae	Pedobacter	kwangyangensis
	5.773	0.015	Proteobacteria	Gammaproteobacteria	Enterobacteriales	Enterobacteriaceae	Citrobacter	freundii
Infant	-5.017	0.091	Firmicutes	Clostridia	Clostridiales	Eubacteriaceae	Eubacterium	callanderi
	-4.930	0.063	Firmicutes	Bacilli	Lactobacillales	Lactobacillaceae	Lactobacillus	taiwanensis
	-4.353	0.081	Bacteroidetes	Bacteroidia	Bacteroidales	Bacteroidaceae	Bacteroides	fragilis
	-2.823	0.083	Bacteroidetes	Bacteroidia	Bacteroidales	Porphyromonadaceae	Parabacteroides	goldsteinii
	-2.572	0.089	Bacteroidetes	Bacteroidia	Bacteroidales	Bacteroidaceae	Bacteroides	
	-2.317	0.089	Bacteroidetes	Bacteroidia	Bacteroidales	Bacteroidaceae	Bacteroides	paurosaccharolyticus
	2.625	0.096	Firmicutes	Bacilli	Bacillales	Bacillaceae	Pontibacillus	halophilus
	2.875	0.091	Actinobacteria	Actinobacteria	Actinomycetales	Actinomycetaceae	Actinomyces	lingnae
	2.969	0.055	Firmicutes	Clostridia	Clostridiales	Clostridiaceae	Clostridium	cavendishii
	3.340	0.063	Proteobacteria	Gammaproteobacteria	Pasteurellales	Pasteurellaceae	Mannheimia	caviae
	3.341	0.091	Proteobacteria	Gammaproteobacteria	Pasteurellales	Pasteurellaceae		
	4.393	0.096	Proteobacteria	Gammaproteobacteria	Pasteurellales	Pasteurellaceae	Actinobacillus	
	4.517	0.096	Firmicutes	Clostridia	Clostridiales	Clostridiaceae	Clostridium	perfringens
	4.714	0.083	Proteobacteria	Gammaproteobacteria	Pasteurellales	Pasteurellaceae	Actinobacillus	rossii
	4.808	0.089	Proteobacteria	Gammaproteobacteria	Pasteurellales	Pasteurellaceae	Actinobacillus	porcinus
	5.491	0.010	Proteobacteria	Gammaproteobacteria	Pasteurellales	Pasteurellaceae	Haemophilus	parainfluenzae

Appendix Table S2: Summary of number of sequenced and classified reads per sample. Classified reads reported have been filtered to remove rare, i.e. noisy, taxa. We omitted noisy taxa found in less than 7 of samples. This threshold was chosen to exclude taxa that do not constitute a “core” microbiome, which suggests a taxon is persistent within at least two mothers throughout the study or that a taxon during development is found in at least 20% of infants at one visit.

Read classification tool	Group	Treatment	Median High Quality Reads	High Quality Reads SD	Reads classified at the genus level	Genus level reads SD	Unique genera identified	Genera identified SD	Percent reads classified at the genus level
**BaseSpace RDP**	**All**	**All**	**47135**	**22116**	**44061**	**21435**	**110**	**31**	**93.53**
	Infant	nTC	47860.5	36374	45598.5	35500	89.5	29	96.07
	Infant	TC	47135	12649	44760	11849	85	25	95.77
	Mother	nTC	46976	11743	43256	10655	124	22	91.31
	Mother	TC	46581	13649	42814	12940	126	25	92.65
**DADA2**	**All**	**All**	**19390**	**10589**	**15498**	**9411**	**18**	**12**	**84.46**
	Infant	nTC	19266	13606	16043	12781	12	5	94.25
	Infant	TC	17653	9121	14489.5	8245	12	5	89.4
	Mother	nTC	20461	9101	15802	7295	34	9	81.65
	Mother	TC	19858	9594	15139.5	7887	33.5	10	81.81
