# Supplementary material for: Household triclosan and triclocarban effects on the infant and maternal microbiome
Source: EMBO Mol Med. 2017 Oct 13;9(12):1732–41. doi: 10.15252/emmm.201707882 (PMC5709730; doi:10.15252/emmm.201707882)
Supplement: Supplementary file 2 — Expanded View Figures PDF [file EMMM-9-1732-s002.pdf]

Expanded View Figures

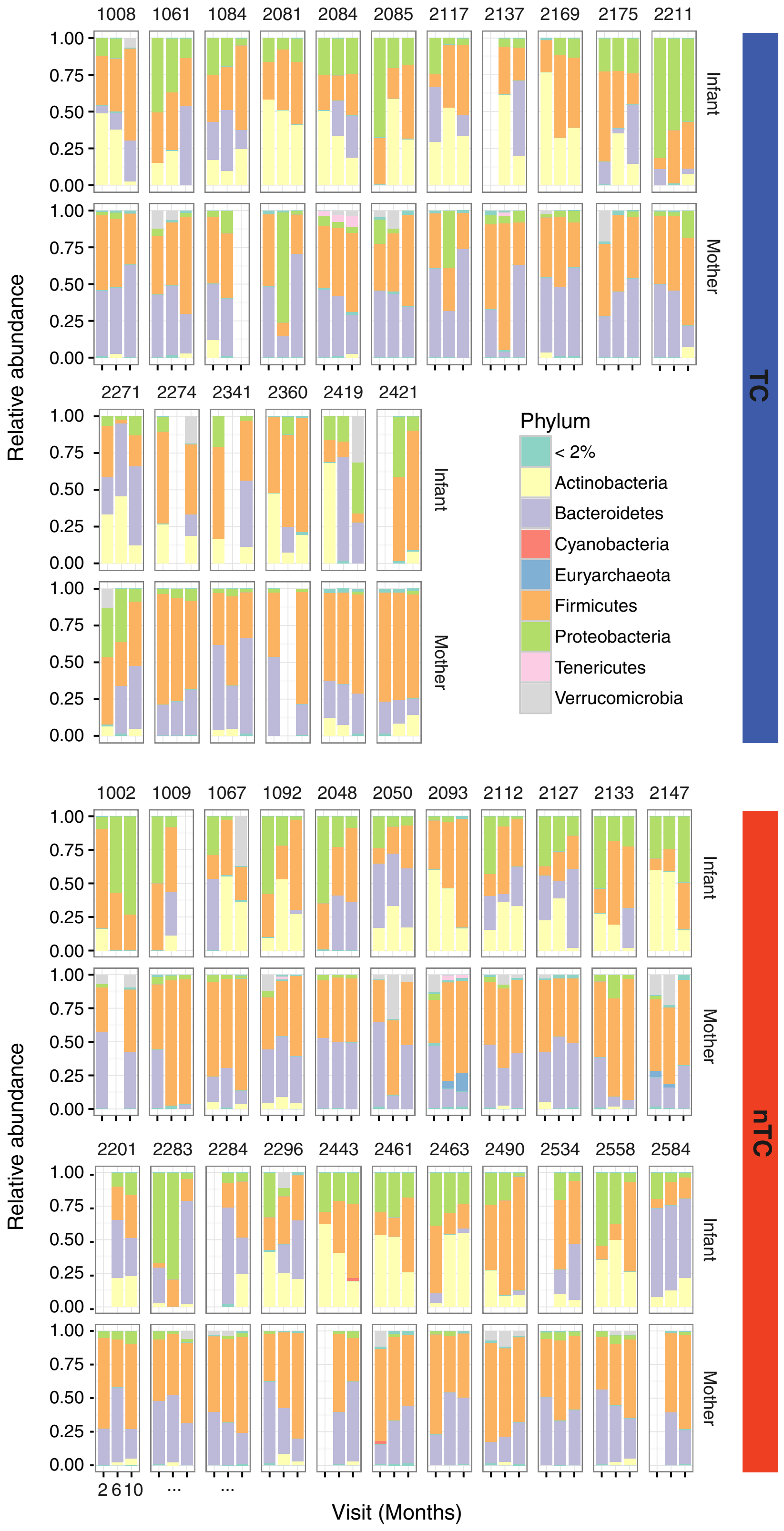

Figure EV1. Individuals in mother and infant groups have similar relative microbiome compositions at the phylum level throughout the first year of life independent of TC exposure.

TC households (n = 17) and nTC (n = 22) households have stable relative abundance of phyla at 2-, 6-, and 10-month visits. Phyla present in < 2% abundance are condensed for visual clarity.

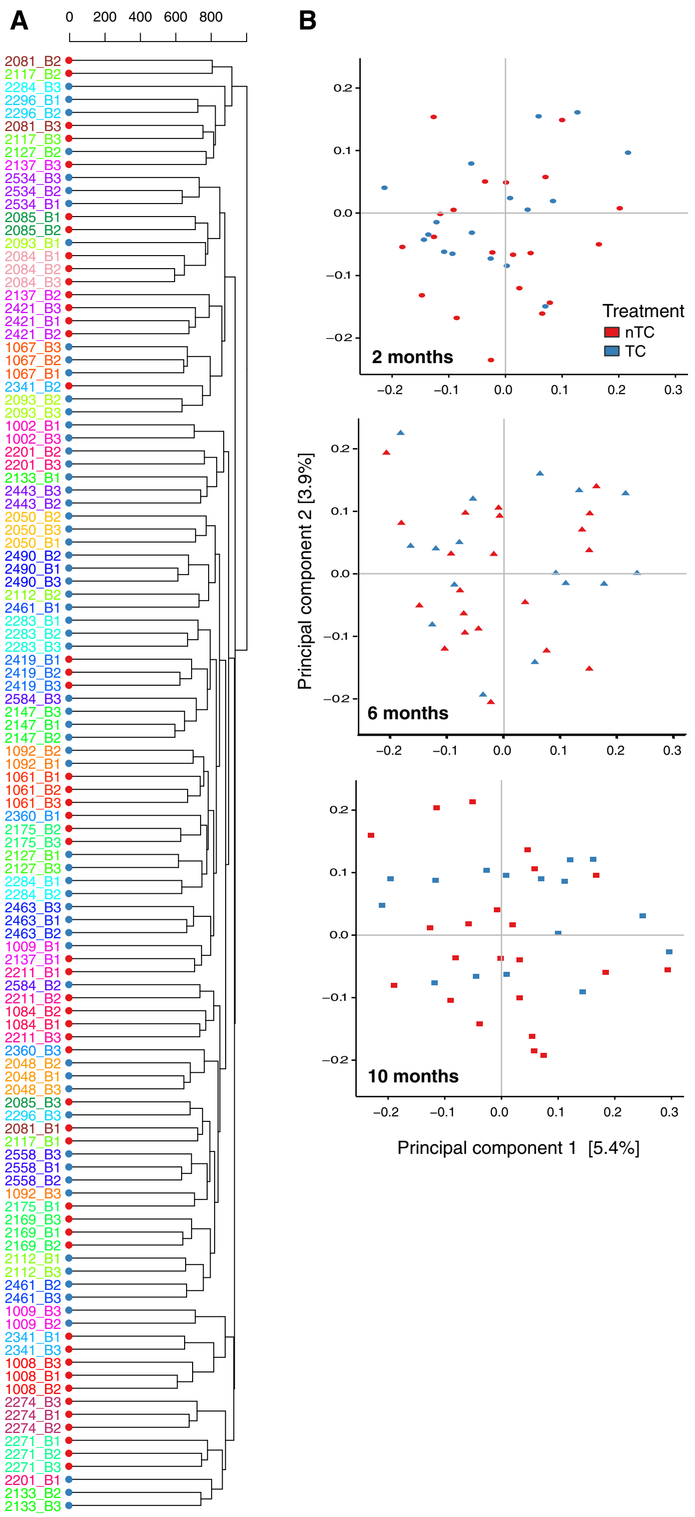

Figure EV2. Maternal samples within households are more similar than between households.

A Hierarchical clustering of Canberra distances shows that maternal samples from a given individual throughout the first year of life are more self-similar than other household mothers at the same visit. The colors are representative of a single household, labeled by household and visit (B1 = 2 months, B2 = 6 months, B3 = 10 months), and node colors reflect TC grouping (red = nTC, blue = TC).

B PCoA of Canberra distances shows 9.3% of the variance is explained by the first two principal components and the absence of TC treatment clustering for any visit among mothers.

 © 2017 The Authors

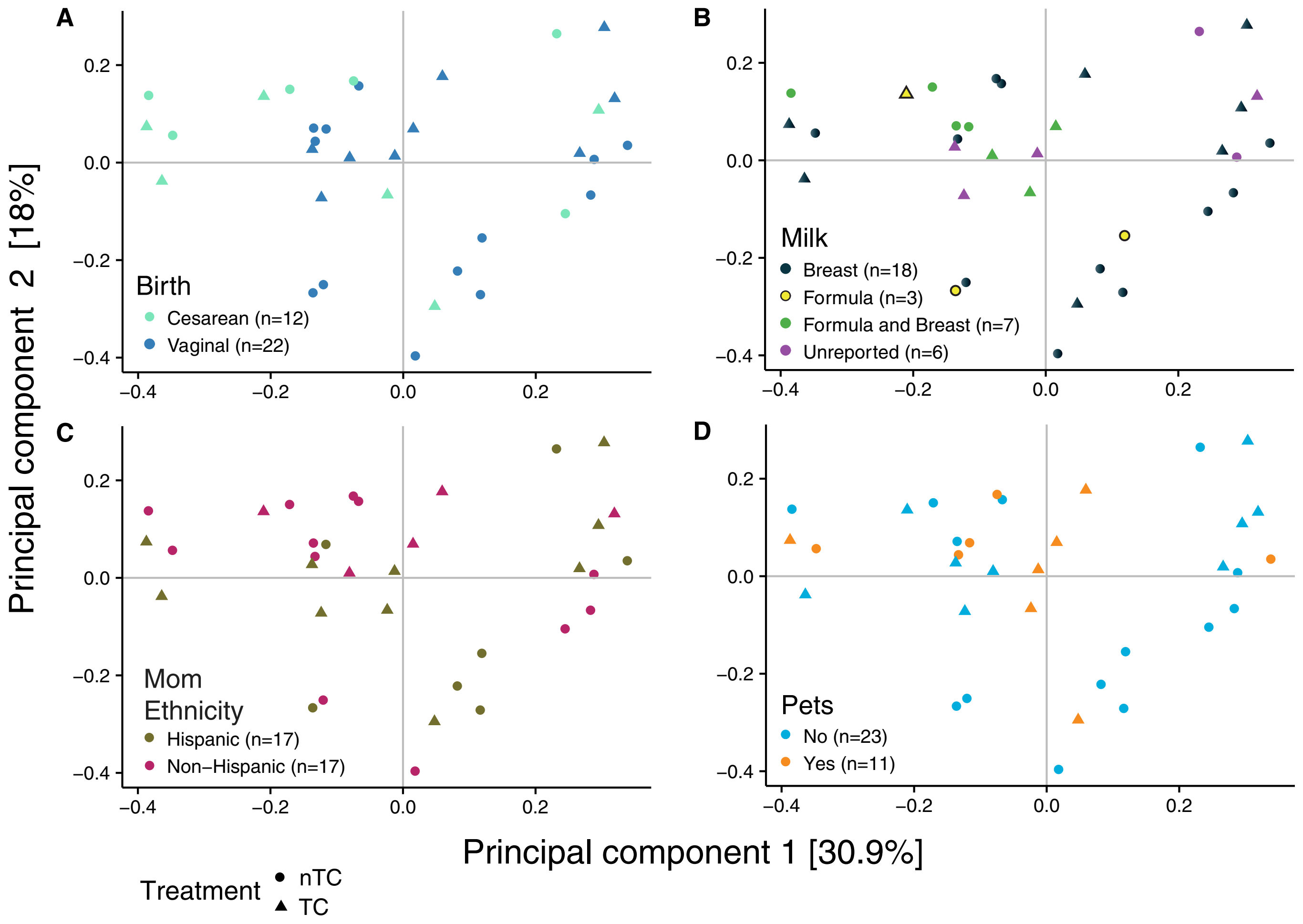

Figure EV3. Infant intestinal microbiome variability is minimally driven by some known external factors by 2 months of age (*n* = 34).

A–D PCoA with Bray–Curtis dissimilarity of the infants at 2 months of age suggests factors known to impact the microbiome, such as (A) birth method (PERMANOVA R^2 = 0.0683, P = 0.011) and (B) formula (R^2 = 0.128, P = 0.041) drive minimal variation. However, (C) maternal ethnicity (R^2 = 0.0274, P = 0.505) and (D) pets (R^2 = 0.0310, P = 0.393) in the household are not sufficient to distinguish the infants at 2 months of age.

© 2017 The Authors

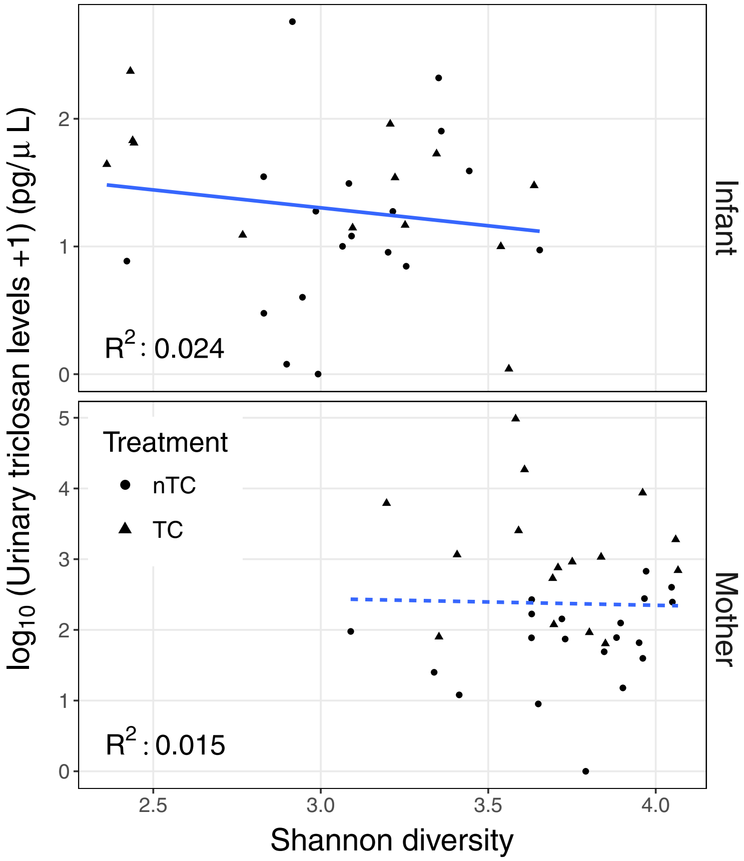

Figure EV4. Microbial diversity does not correlate with urinary triclosan levels at 6 months for mothers or infants.

Correlations were determined using a linear regression model (Shannon diversity ~ Urinary triclosan levels) on mother and infant samples separately.

 © 2017 The Authors

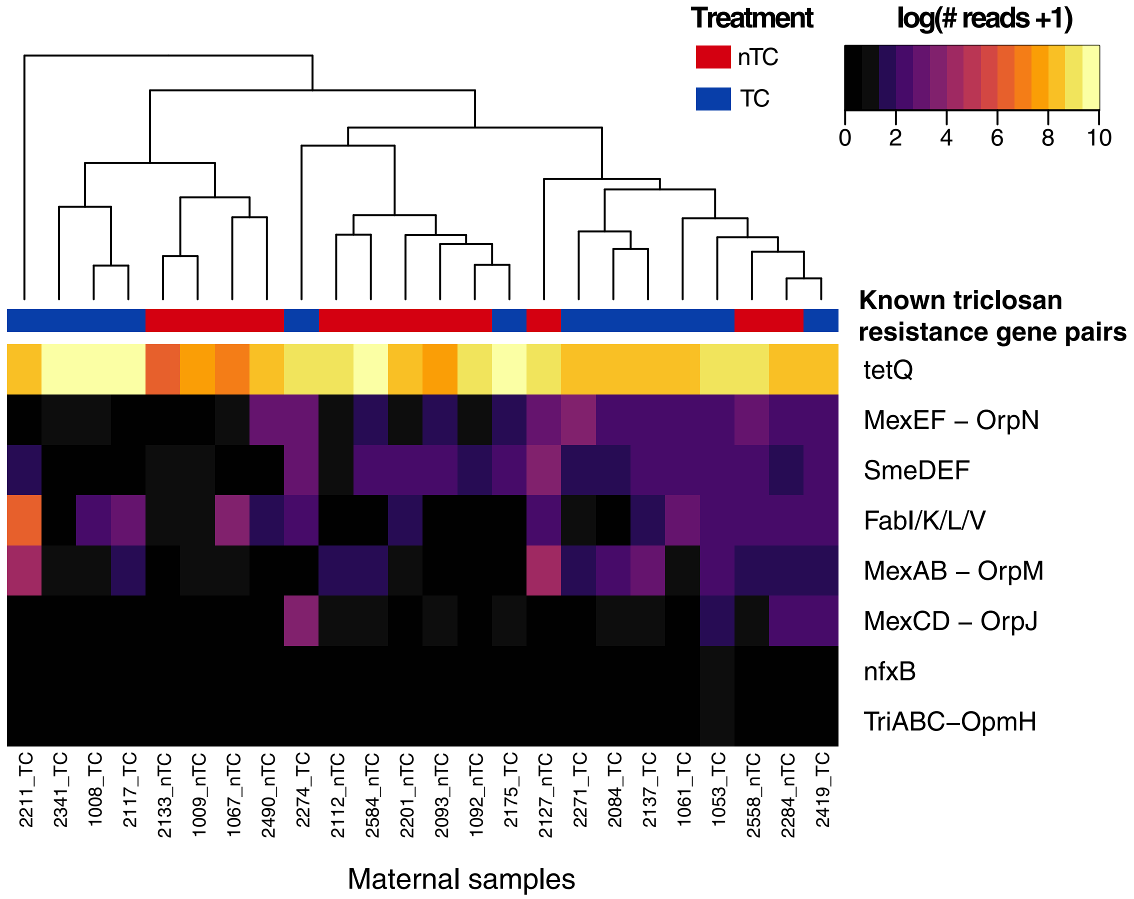

Figure EV5. Triclosan resistance gene abundances do not distinguish TC and nTC maternal samples following 6 months of exposure.

Euclidean distance was calculated between samples of whole shotgun sequencing reads that aligned to CARD (approximately 0.03% or 6,000 reads for each sample in both intervention arms), then clustered with a hierarchical agglomeration method.

© 2017 The Authors
