# Supplementary material for: Household triclosan and triclocarban effects on the infant and maternal microbiome
Source: EMBO Mol Med. 2017 Oct 13;9(12):1732–41. doi: 10.15252/emmm.201707882 (PMC5709730; doi:10.15252/emmm.201707882)
Supplement: Supplementary file 4 — Source Data for Figure 1 [file EMMM-9-1732-s003.pdf]

Household	Treatment	Group	TC Level (pg/mL)	Group	TC Level (pg/mL)
**All**	**TC**	**Infant**	**43.00 (0.1-234.8)**	**Mother**	**916.10 (63-96664.5)**
1008	TC	Infant	29	Mother	18502
1061	TC	Infant	52.2	Mother	96664.5
1084	TC	Infant	NA	Mother	91
2081	TC	Infant	33.6	Mother	78.7
2084	TC	Infant	0.1	Mother	693.6
2085	TC	Infant	63.7	Mother	1070.9
2117	TC	Infant	11.3	Mother	916.1
2137	TC	Infant	NA	Mother	118
2169	TC	Infant	234.8	Mother	1156.4
2175	TC	Infant	9	Mother	63
2211	TC	Infant	90	Mother	758
2271	TC	Infant	43	Mother	2538
2274	TC	Infant	84.6	Mother	6177
2341	TC	Infant	52.4	Mother	534.9
2360	TC	Infant	13.7	Mother	123.4
2419	TC	Infant	66.6	Mother	8681.4
2421	TC	Infant	13	Mother	1896
**All**	**nTC**	**Infant**	**10.05 (0 - 574.2)**	**Mother**	**76.00 (0 - 677.1)**
1002	nTC	Infant	6	Mother	15
1009	nTC	Infant	NA	Mother	11
1067	nTC	Infant	574.2	Mother	48
1092	nTC	Infant	2	Mother	399.3
2048	nTC	Infant	17.9	Mother	141.5
2050	nTC	Infant	NA	Mother	24
2093	nTC	Infant	79	Mother	NA
2112	nTC	Infant	9	Mother	65
2127	nTC	Infant	38	Mother	268
2133	nTC	Infant	17.8	Mother	76
2147	nTC	Infant	11.1	Mother	76.8
2201	nTC	Infant	8	Mother	73
2283	nTC	Infant	0.2	Mother	247.6
2284	nTC	Infant	6.7	Mother	14.1
2296	nTC	Infant	8.4	Mother	166.9
2443	nTC	Infant	3	Mother	94
2461	nTC	Infant	34.2	Mother	677.1
2463	nTC	Infant	30.1	Mother	123.5
2490	nTC	Infant	207.7	Mother	0
2534	nTC	Infant	NA	Mother	276
2558	nTC	Infant	0	Mother	38.4
2584	nTC	Infant	NA	Mother	8
