# Supplementary material for: Household triclosan and triclocarban effects on the infant and maternal microbiome
Source: EMBO Mol Med. 2017 Oct 13;9(12):1732–41. doi: 10.15252/emmm.201707882 (PMC5709730; doi:10.15252/emmm.201707882)
Supplement: Supplementary file 5 — Source Data for Figure 2 [file EMMM-9-1732-s004.pdf]

| Household | Visit | Group | PC1 | PC2 |
|---|---|---|---|---|
| 1008 | 2 | Mother | 0.270003011 | 0.104281825 |
| 1008 | 2 | Infant | -0.211946457 | 0.185220215 |
| 1061 | 2 | Mother | 0.23779819 | -0.100977876 |
| 1061 | 2 | Infant | -0.304420674 | 0.062986133 |
| 1067 | 2 | Infant | -0.113866799 | -0.249456394 |
| 1067 | 2 | Mother | 0.184964304 | 0.187733453 |
| 1092 | 2 | Infant | -0.396910106 | 0.002600567 |
| 1092 | 2 | Mother | 0.267284956 | -0.08980873 |
| 2048 | 2 | Infant | -0.212927535 | -0.298985195 |
| 2048 | 2 | Mother | 0.272760896 | -0.137251437 |
| 2050 | 2 | Infant | -0.247573169 | -0.21370144 |
| 2050 | 2 | Mother | 0.163408419 | -0.133280518 |
| 2081 | 2 | Infant | -0.523645407 | 0.162380408 |
| 2081 | 2 | Mother | 0.306909508 | 0.0803721 |
| 2084 | 2 | Mother | 0.261645583 | -0.012387669 |
| 2084 | 2 | Infant | -0.393998984 | -0.092896643 |
| 2085 | 2 | Infant | -0.234961074 | -0.295362089 |
| 2085 | 2 | Mother | 0.133114615 | -0.151316326 |
| 2093 | 2 | Infant | -0.340460021 | 0.188703518 |
| 2093 | 2 | Mother | 0.198867515 | -0.143007398 |
| 2112 | 2 | Infant | -0.214606811 | -0.169803423 |
| 2112 | 2 | Mother | 0.223593729 | -0.115650778 |
| 2117 | 2 | Infant | -0.332609374 | -0.233073499 |
| 2117 | 2 | Mother | 0.269679616 | -0.002325895 |
| 2127 | 2 | Infant | -0.365641114 | -0.166021369 |
| 2127 | 2 | Mother | 0.290441551 | 0.042187726 |
| 2133 | 2 | Infant | -0.35662975 | -0.202923922 |
| 2133 | 2 | Mother | 0.298726381 | -0.017162541 |
| 2147 | 2 | Infant | -0.518240781 | 0.148176493 |
| 2147 | 2 | Mother | 0.266284403 | 0.052010111 |
| 2169 | 2 | Infant | -0.449778159 | 0.197930567 |
| 2169 | 2 | Mother | 0.193257771 | -0.100560437 |
| 2175 | 2 | Infant | -0.198989989 | -0.252583847 |
| 2175 | 2 | Mother | 0.232674856 | 0.024606624 |
| 2271 | 2 | Infant | -0.253916519 | -0.045273668 |
| 2271 | 2 | Mother | -0.044945264 | 0.129863938 |
| 2274 | 2 | Infant | -0.179162128 | 0.02861391 |
| 2274 | 2 | Mother | 0.152471217 | 0.027964893 |
| 2283 | 2 | Infant | -0.216784002 | -0.239016892 |
| 2283 | 2 | Mother | 0.244836187 | 0.010941242 |
| 2284 | 2 | Mother | 0.306989697 | 0.004810727 |
| 2296 | 2 | Infant | -0.390314765 | 0.090295447 |
| 2296 | 2 | Mother | 0.140073098 | -0.203110927 |
| 2341 | 2 | Infant | -0.412381073 | 0.06632838 |
| 2341 | 2 | Mother | 0.187209518 | 0.066783071 |
| 2360 | 2 | Infant | -0.179564613 | 0.00110587 |
| 2360 | 2 | Mother | 0.29818939 | -0.076324078 |

| | | | | |
|---|---|---|---|---|
| 2419 | 2 | Infant | -0.507904968 | 0.179215142 |
| 2419 | 2 | Mother | 0.223052868 | 0.066305826 |
| 2461 | 2 | Infant | -0.534168704 | 0.15276312 |
| 2461 | 2 | Mother | 0.21222286 | 0.091692554 |
| 2463 | 2 | Infant | -0.23042511 | 0.031315966 |
| 2463 | 2 | Mother | 0.300085589 | 0.099407553 |
| 2490 | 2 | Infant | -0.233816654 | 0.047363997 |
| 2490 | 2 | Mother | 0.278210739 | 0.14943659 |
| 2558 | 2 | Infant | -0.508176859 | 0.104035631 |
| 2558 | 2 | Mother | 0.225275606 | -0.125313231 |
| 2584 | 2 | Infant | -0.083977196 | -0.238901682 |
| 2211 | 2 | Infant | -0.22603731 | -0.311596041 |
| 1002 | 2 | Mother | 0.254664403 | -0.144461923 |
| 2443 | 2 | Infant | -0.507980805 | 0.166720908 |
| 1009 | 2 | Mother | 0.274736084 | -0.050167578 |
| 2201 | 2 | Mother | 0.182975943 | 0.042004837 |
| 2137 | 2 | Mother | 0.272624369 | 0.075188445 |
| 1009 | 2 | Infant | -0.2379295 | -0.30990275 |
| 1084 | 2 | Mother | 0.211941053 | -0.011008141 |
| 2211 | 2 | Mother | 0.283554442 | 0.003711857 |
| 1084 | 2 | Infant | -0.17589403 | -0.136561497 |
| 1002 | 2 | Infant | -0.049636354 | 0.118442095 |
| 2534 | 2 | Mother | 0.290749192 | 0.043640823 |
| 2421 | 2 | Mother | 0.282648964 | 0.114309228 |
| 1008 | 6 | Infant | -0.231566283 | 0.139412349 |
| 1008 | 6 | Mother | 0.232242905 | 0.077719428 |
| 1061 | 6 | Infant | -0.334091921 | -0.01454174 |
| 1061 | 6 | Mother | 0.29829984 | -0.098251297 |
| 1067 | 6 | Infant | -0.391050192 | 0.156135417 |
| 1067 | 6 | Mother | 0.293920817 | 0.13729463 |
| 1092 | 6 | Infant | -0.467859648 | 0.121694246 |
| 1092 | 6 | Mother | 0.23956855 | -0.058715422 |
| 2048 | 6 | Infant | -0.205717032 | -0.139893332 |
| 2048 | 6 | Mother | 0.239679864 | -0.109951638 |
| 2050 | 6 | Infant | -0.251940918 | -0.129671834 |
| 2050 | 6 | Mother | 0.2084705 | 0.085259829 |
| 2081 | 6 | Infant | -0.471688686 | 0.152996938 |
| 2081 | 6 | Mother | -0.160840841 | -0.068662419 |
| 2084 | 6 | Infant | -0.309534415 | -0.176497278 |
| 2084 | 6 | Mother | 0.242872388 | 0.022551281 |
| 2085 | 6 | Infant | -0.24576672 | -0.159328746 |
| 2085 | 6 | Mother | 0.270542397 | -0.100197856 |
| 2093 | 6 | Infant | -0.289345172 | 0.191541829 |
| 2093 | 6 | Mother | 0.241899966 | 0.162032918 |
| 2112 | 6 | Infant | -0.304032998 | 0.104215905 |
| 2112 | 6 | Mother | 0.239120761 | 0.071457858 |
| 2117 | 6 | Infant | -0.396472795 | 0.132413102 |
| 2117 | 6 | Mother | 0.039337494 | -0.066086856 |

| | | | | |
|---|---|---|---|---|
| 2127 | 6 | Infant | -0.353888106 | 0.068830663 |
| 2127 | 6 | Mother | 0.220256868 | -0.015709975 |
| 2133 | 6 | Infant | -0.334746315 | 0.112471853 |
| 2133 | 6 | Mother | 0.179337283 | 0.049606059 |
| 2147 | 6 | Infant | -0.303400691 | 0.030278808 |
| 2147 | 6 | Mother | 0.272457249 | 0.07809782 |
| 2169 | 6 | Infant | -0.415963606 | 0.113819762 |
| 2169 | 6 | Mother | 0.215820947 | -0.099279985 |
| 2175 | 6 | Infant | -0.427170036 | 0.008813128 |
| 2175 | 6 | Mother | 0.317897118 | -0.00231552 |
| 2271 | 6 | Infant | -0.271303947 | -0.030676746 |
| 2271 | 6 | Mother | 0.093035156 | -0.197630793 |
| 2274 | 6 | Mother | 0.083902321 | -0.030832766 |
| 2283 | 6 | Infant | -0.24089653 | -0.309803245 |
| 2283 | 6 | Mother | 0.240794008 | -0.009284447 |
| 2284 | 6 | Infant | -0.105573337 | -0.223733848 |
| 2284 | 6 | Mother | 0.28145933 | -0.005100593 |
| 2296 | 6 | Infant | -0.174580734 | -0.078498897 |
| 2296 | 6 | Mother | 0.240121128 | 0.165264947 |
| 2341 | 6 | Mother | 0.153360318 | 0.147226767 |
| 2360 | 6 | Infant | -0.189399342 | -0.013611527 |
| 2419 | 6 | Infant | -0.096433019 | -0.301706135 |
| 2419 | 6 | Mother | 0.265510332 | 0.105990625 |
| 2461 | 6 | Infant | -0.502994822 | 0.131026415 |
| 2461 | 6 | Mother | 0.306707551 | 0.015239466 |
| 2463 | 6 | Infant | -0.462771843 | -0.010666108 |
| 2463 | 6 | Mother | 0.316092552 | -0.082962284 |
| 2490 | 6 | Infant | -0.122327314 | 0.067673657 |
| 2490 | 6 | Mother | 0.261029317 | 0.128731847 |
| 2558 | 6 | Infant | -0.50020651 | 0.128535521 |
| 2558 | 6 | Mother | 0.234737711 | -0.100514844 |
| 2584 | 6 | Mother | 0.288130301 | -0.014576033 |
| 2534 | 6 | Infant | -0.241755809 | 0.002641222 |
| 2534 | 6 | Mother | 0.256073856 | 0.062829156 |
| 2584 | 6 | Infant | -0.040920046 | -0.203094645 |
| 1009 | 6 | Infant | -0.103543899 | -0.003551644 |
| 2137 | 6 | Mother | 0.242139412 | 0.178125337 |
| 2137 | 6 | Infant | -0.445106308 | 0.160516631 |
| 2211 | 6 | Infant | -0.189702749 | -0.264391951 |
| 1002 | 6 | Infant | -0.135977203 | -0.232992904 |
| 2443 | 6 | Infant | -0.470023461 | 0.150431461 |
| 2211 | 6 | Mother | 0.277585971 | -0.043627166 |
| 2201 | 6 | Infant | -0.311165441 | -0.050846286 |
| 2201 | 6 | Mother | 0.197146387 | -0.160277921 |
| 1084 | 6 | Infant | -0.064125583 | -0.239672419 |
| 1084 | 6 | Mother | 0.234158354 | -0.199034677 |
| 1009 | 6 | Mother | 0.200846857 | 0.200380815 |
| 2421 | 6 | Infant | -0.23023196 | -0.265118537 |

| | | | | |
|---|---|---|---|---|
| 2421 | 6 | Mother | 0.23202076 | 0.158938212 |
| 2443 | 6 | Mother | 0.213001761 | 0.036090942 |
| 1008 | 10 | Infant | 0.206809579 | -0.010097979 |
| 1008 | 10 | Mother | 0.234737899 | 0.05020932 |
| 1061 | 10 | Infant | -0.087994336 | -0.152234781 |
| 1061 | 10 | Mother | 0.310883774 | 0.065689594 |
| 1067 | 10 | Infant | -0.183398611 | 0.145780722 |
| 1067 | 10 | Mother | 0.179302772 | 0.202859947 |
| 1092 | 10 | Infant | -0.209488679 | 0.143923454 |
| 1092 | 10 | Mother | 0.24371969 | 0.022907375 |
| 2048 | 10 | Infant | -0.131047956 | -0.149952425 |
| 2048 | 10 | Mother | 0.294154178 | -0.128971424 |
| 2050 | 10 | Infant | -0.167769907 | -0.164323388 |
| 2050 | 10 | Mother | 0.309311155 | 0.003460732 |
| 2081 | 10 | Infant | -0.39101873 | 0.087961434 |
| 2081 | 10 | Mother | 0.179480165 | -0.004258483 |
| 2084 | 10 | Infant | -0.181143208 | -0.24318498 |
| 2084 | 10 | Mother | 0.257484299 | 0.08200526 |
| 2085 | 10 | Infant | -0.274723608 | 0.151360434 |
| 2085 | 10 | Mother | 0.311476029 | 0.02893741 |
| 2093 | 10 | Infant | 0.076261464 | 0.274484075 |
| 2093 | 10 | Mother | 0.218187043 | 0.145814265 |
| 2112 | 10 | Infant | 0.007913976 | 0.125713247 |
| 2112 | 10 | Mother | 0.282796219 | -0.000121364 |
| 2117 | 10 | Infant | -0.061376938 | 0.101358801 |
| 2117 | 10 | Mother | 0.189863583 | -0.003403967 |
| 2127 | 10 | Infant | -0.025714379 | -0.08914449 |
| 2127 | 10 | Mother | 0.319747132 | 0.040441642 |
| 2133 | 10 | Infant | -0.061545175 | 0.083416724 |
| 2133 | 10 | Mother | 0.243204408 | 0.124407414 |
| 2147 | 10 | Infant | -0.45333291 | 0.002718184 |
| 2147 | 10 | Mother | 0.326666606 | 0.034419716 |
| 2169 | 10 | Infant | -0.420890292 | 0.13698287 |
| 2169 | 10 | Mother | 0.191484105 | -0.18035023 |
| 2175 | 10 | Infant | -0.236744456 | -0.165275275 |
| 2175 | 10 | Mother | 0.280976358 | -0.07712417 |
| 2271 | 10 | Infant | -0.070766011 | -0.074234237 |
| 2271 | 10 | Mother | 0.160869702 | -0.100041881 |
| 2274 | 10 | Infant | 0.008366954 | 0.208882099 |
| 2274 | 10 | Mother | 0.150514452 | -0.033326623 |
| 2283 | 10 | Infant | 0.047388166 | -0.106499238 |
| 2283 | 10 | Mother | 0.297160981 | 0.065585938 |
| 2284 | 10 | Infant | -0.111575862 | 0.103548301 |
| 2284 | 10 | Mother | 0.092518294 | -0.005568702 |
| 2296 | 10 | Infant | 0.074907207 | -0.001783577 |
| 2296 | 10 | Mother | 0.220725293 | 0.148678303 |
| 2341 | 10 | Infant | 0.014033986 | 0.073713244 |
| 2341 | 10 | Mother | 0.191493065 | 0.032742671 |

| | | | | |
|---|---|---|---|---|
| 2360 | 10 | Infant | -0.127001676 | 0.171339246 |
| 2360 | 10 | Mother | 0.251636356 | 0.086406877 |
| 2419 | 10 | Infant | -0.080966879 | -0.434359343 |
| 2419 | 10 | Mother | 0.313697142 | 0.028847697 |
| 2461 | 10 | Infant | -0.46173005 | 0.086939976 |
| 2461 | 10 | Mother | 0.309621587 | -0.059006641 |
| 2463 | 10 | Infant | -0.45930901 | 0.126612001 |
| 2463 | 10 | Mother | 0.284302263 | -0.027615238 |
| 2490 | 10 | Infant | 0.00571946 | 0.189777384 |
| 2490 | 10 | Mother | 0.318675829 | 0.06530859 |
| 2558 | 10 | Infant | -0.270902575 | 0.110749434 |
| 2558 | 10 | Mother | 0.260112175 | 0.025668845 |
| 1002 | 10 | Infant | -0.225152575 | -0.026450248 |
| 2584 | 10 | Infant | -0.094641744 | -0.171958493 |
| 2584 | 10 | Mother | 0.305118886 | 0.048288449 |
| 2443 | 10 | Infant | -0.364495337 | 0.072861754 |
| 2443 | 10 | Mother | 0.236489896 | -0.174214703 |
| 2534 | 10 | Infant | 0.090448697 | 0.061445442 |
| 2534 | 10 | Mother | 0.221951076 | 0.064387062 |
| 2421 | 10 | Mother | 0.138881814 | 0.195879617 |
| 1009 | 10 | Mother | 0.226650754 | 0.18466187 |
| 2137 | 10 | Infant | 0.033376839 | 0.085049739 |
| 1084 | 10 | Infant | -0.023882965 | 0.128362274 |
| 1002 | 10 | Mother | 0.261864742 | -0.013533425 |
| 2137 | 10 | Mother | 0.228722677 | 0.010111221 |
| 2201 | 10 | Infant | -0.236255442 | -0.00700493 |
| 2211 | 10 | Mother | 0.125709866 | 0.138950627 |
| 2211 | 10 | Infant | -0.263264463 | -0.026095439 |
| 2421 | 10 | Infant | 0.084769975 | 0.17765757 |
| 2201 | 10 | Mother | 0.176778643 | 0.056574345 |
