# Supplementary material for: Household triclosan and triclocarban effects on the infant and maternal microbiome
Source: EMBO Mol Med. 2017 Oct 13;9(12):1732–41. doi: 10.15252/emmm.201707882 (PMC5709730; doi:10.15252/emmm.201707882)
Supplement: Supplementary file 6 — Source Data for Figure 3 [file EMMM-9-1732-s005.pdf]

Household	Group	Treatment	2 months	6 months	10 months	
**All**	**Infant**	**nTC**	**2.89**	**3.0935**	**3.264**	(Medians)
**All**	**Group**	**nTC**	**3.8075**	**3.846**	**3.796**	
1002	Infant	nTC	3.543	3.255	3.201	
1009	Infant	nTC	2.89	3.158	NA	
1067	Infant	nTC	2.94	2.916	2.612	
1092	Infant	nTC	2.863	2.83	3.366	
2048	Infant	nTC	2.676	2.985	2.898	
2050	Infant	nTC	3.252	3.095	3.472	
2093	Infant	nTC	2.792	3.36	3.405	
2112	Infant	nTC	3.519	3.065	3.407	
2127	Infant	nTC	3.024	3.443	2.964	
2133	Infant	nTC	2.692	3.215	2.963	
2147	Infant	nTC	2.646	3.092	3.392	
2201	Infant	nTC	NA	3.201	3.551	
2283	Infant	nTC	2.437	2.899	2.168	
2284	Infant	nTC	NA	2.421	3.215	
2296	Infant	nTC	2.979	3.654	3.66	
2443	Infant	nTC	2.792	2.946	3.299	
2461	Infant	nTC	2.67	2.829	3.264	
2463	Infant	nTC	2.938	3.084	3.1	
2490	Infant	nTC	3.139	3.352	3.409	
2534	Infant	nTC	NA	3.525	2.869	
2558	Infant	nTC	2.738	2.992	3.233	
2584	Infant	nTC	3.042	3.218	3.424	
1002	Mother	nTC	3.666	NA	3.682	
1009	Mother	nTC	3.976	3.414	3.519	
1067	Mother	nTC	3.854	3.846	3.836	
1092	Mother	nTC	3.917	4.047	3.619	
2048	Mother	nTC	3.551	3.72	3.756	
2050	Mother	nTC	3.453	3.339	3.622	
2093	Mother	nTC	3.852	4.043	3.994	
2112	Mother	nTC	3.777	3.95	3.869	
2127	Mother	nTC	4.193	3.63	3.697	
2133	Mother	nTC	3.714	3.629	3.404	
2147	Mother	nTC	3.928	3.883	4.047	
2201	Mother	nTC	3.512	3.729	3.997	
2283	Mother	nTC	3.87	4.05	3.939	
2284	Mother	nTC	3.798	3.902	3.739	
2296	Mother	nTC	3.193	3.63	3.688	
2443	Mother	nTC	NA	3.089	3.442	
2461	Mother	nTC	3.817	3.971	3.867	
2463	Mother	nTC	4.002	3.895	3.951	
2490	Mother	nTC	3.696	3.791	3.877	
2534	Mother	nTC	3.829	3.966	3.744	
2558	Mother	nTC	3.629	3.962	3.877	
2584	Mother	nTC	NA	3.649	4.001	

**All**	**Infant**	**TC**	**2.913**	**3.207**	**3.26**
**All**	**Group**	**TC**	**3.713**	**3.702**	**3.565**
1008	Infant	TC	3.377	3.637	3.655
1061	Infant	TC	2.871	3.346	3.225
1084	Infant	TC	3.817	3.913	3.805
2081	Infant	TC	2.963	3.222	3.486
2084	Infant	TC	2.913	3.562	3.717
2085	Infant	TC	2.825	2.442	3.605
2117	Infant	TC	2.985	2.767	3.26
2137	Infant	TC	NA	3.042	3.292
2169	Infant	TC	2.566	2.432	2.641
2175	Infant	TC	3.295	3.538	3.466
2211	Infant	TC	2.636	3.207	3.437
2271	Infant	TC	3.19	2.361	3.094
2274	Infant	TC	3.303	NA	3.135
2341	Infant	TC	2.778	NA	2.878
2360	Infant	TC	2.682	3.252	3.249
2419	Infant	TC	2.788	2.438	2.553
2421	Infant	TC	NA	3.095	3.143
1008	Mother	TC	3.445	3.608	2.92
1061	Mother	TC	3.909	3.582	3.961
1084	Mother	TC	3.971	3.802	NA
2081	Mother	TC	3.329	3.353	2.353
2084	Mother	TC	3.934	4.067	4.289
2085	Mother	TC	3.885	3.837	3.857
2117	Mother	TC	3.339	3.751	2.572
2137	Mother	TC	3.977	3.696	3.094
2169	Mother	TC	3.437	3.407	3.264
2175	Mother	TC	3.713	3.85	3.872
2211	Mother	TC	3.647	3.708	3.951
2271	Mother	TC	3.681	3.591	3.547
2274	Mother	TC	3.363	3.196	3.583
2341	Mother	TC	3.081	3.692	3.023
2360	Mother	TC	3.881	NA	3.35
2419	Mother	TC	4.137	3.961	4.102
2421	Mother	TC	4.087	4.06	4.024
