# Supplementary material for: Household triclosan and triclocarban effects on the infant and maternal microbiome
Source: EMBO Mol Med. 2017 Oct 13;9(12):1732–41. doi: 10.15252/emmm.201707882 (PMC5709730; doi:10.15252/emmm.201707882)
Supplement: Supplementary file 7 — Source Data for Figure 4 [file EMMM-9-1732-s006.zip › Source_Data_figure4/EMM-2017-07882-SourceDataForFigure4A.pdf]

Group	Enrichment	Log2 Fold Change	FDR adjusted p-value	Phylum	Class	Order	Family	Genus	Species
**Infant**	**nTC**	2.553	0.0038	Firmicutes	Clostridia	Clostridiales	Clostridiaceae	Clostridium	paraputrificum
		2.62	0.0003	Proteobacteria	Gammaproteobacteria	Enterobacteriales	Enterobacteriaceae	Serratia	marcescens
		4.3	0.0003	Bacteroidetes	Bacteroidia	Bacteroidales	Bacteroidaceae	Bacteroides	fragilis
	**TC**	1.702	0.0062	Firmicutes	Bacilli	Lactobacillales	Enterococcaceae	Enterococcus	
		1.716	0.005	Firmicutes	Bacilli	Lactobacillales	Enterococcaceae	Vagococcus	teuberi
		1.835	0.0084	Firmicutes	Clostridia	Clostridiales	Ruminococcaceae	Ruminococcus	bromii
		2.271	0.0084	Firmicutes	Clostridia	Coriobacteriales	Coriobacteriaceae	Eggerthella	
		2.709	4.61E-06	Actinobacteria	Actinobacteria	Bifidobacteriales	Bifidobacteriaceae	Bifidobacterium	dentium
		2.765	0.001	Proteobacteria	Deltaproteobacteria	Desulfovibrionales	Desulfovibrionaceae	Desulfovibrio	
		3.233	6.17E-09	Verrucomicrobia	Verrucomicrobiae	Verrucomicrobiales	Verrucomicrobiaceae	Akkermansia	muciniphila
		3.268	0.0008	Firmicutes	Clostridia	Clostridiales	Clostridiaceae	Clostridium	cadaveris
		3.355	0.0074	Firmicutes	Clostridia	Clostridiales	Clostridiaceae	Clostridium	perfringens
		3.548	0.0097	Firmicutes	Clostridia	Clostridiales	Lachnospiraceae	Blautia	glucerasea
		3.575	0.0008	Proteobacteria	Betaproteobacteria	Burkholderiales	Alcaligenaceae	Sutterella	stercoricanis
		4.082	0.003	Firmicutes	Bacilli	Lactobacillales	Streptococcaceae	Streptococcus	infantarius
		4.416	0.0004	Firmicutes	Clostridia	Clostridiales	Veillonellaceae	Megasphaera	elsdenii
		0.777	0.0095	Firmicutes	Bacilli	Bacillales	Bacillaceae	Bacillus	
**Mother**	**nTC**	1.562	0.0012	Bacteroidetes	Bacteroidia	Bacteroidales	Porphyromonadaceae	Parabacteroides	goldsteinii
		1.64	0.0037	Firmicutes	Clostridia	Clostridiales	Lachnospiraceae	Butyrivibrio	proteoclasticus
		2.065	1.12E-06	Bacteroidetes	Bacteroidia	Bacteroidales	Bacteroidaceae	Bacteroides	sartorii
		2.326	1.52E-05	Bacteroidetes	Bacteroidia	Bacteroidales	Porphyromonadaceae	Dysgonomonas	wimpennyi
		3.519	5.14E-07	Bacteroidetes	Bacteroidia	Bacteroidales	Bacteroidaceae	Bacteroides	stercoris
		4.388	0.0004	Bacteroidetes	Bacteroidia	Bacteroidales	Bacteroidaceae	Bacteroides	massiliensis
		5.206	1.23E-08	Bacteroidetes	Bacteroidia	Bacteroidales	Bacteroidaceae	Bacteroides	clarus
		5.292	1.31E-09	Bacteroidetes	Bacteroidia	Bacteroidales	Bacteroidaceae	Bacteroides	intestinalis
	**TC**	1.178	0.0011	Bacteroidetes	Sphingobacteriia	Sphingobacteriales	Rhodothermaceae	Rhodothermus	clarus
		1.313	0.0011	Proteobacteria	Gammaproteobacteria	Enterobacteriales	Enterobacteriaceae	Enterobacter	nickellidurans
		1.387	0.0039	Bacteroidetes	Bacteroidia	Bacteroidales			
		1.54	5.16E-05	Firmicutes	Clostridia	Clostridiales	Veillonellaceae	Megasphaera	hominis
		1.858	0.0072	Firmicutes	Clostridia	Clostridiales	Veillonellaceae	Phascolarcto-bacterium	succinatutens
		1.953	0.0056	Proteobacteria	Gammaproteobacteria	Enterobacteriales	Enterobacteriaceae	Serratia	
		2.325	0.0037	Proteobacteria	Gammaproteobacteria	Enterobacteriales	Enterobacteriaceae	Escherichia	albertii
		2.334	0.0021	Proteobacteria	Gammaproteobacteria	Enterobacteriales	Enterobacteriaceae	Trabulsiella	odontotermitis
		2.405	0.0019	Proteobacteria	Gammaproteobacteria	Enterobacteriales	Enterobacteriaceae	Escherichia	
		2.514	0.0001	Proteobacteria	Gammaproteobacteria	Enterobacteriales	Enterobacteriaceae	Enterobacter	amnigenus
		2.528	0.0006	Proteobacteria	Gammaproteobacteria	Aeromonadales	Aeromonadaceae	Tolumonas	auensis
		2.55	0.0056	Bacteroidetes	Bacteroidia	Bacteroidales	Prevotellaceae	Prevotella	copri
		2.552	0.0087	Proteobacteria	Gammaproteobacteria	Enterobacteriales	Enterobacteriaceae	Escherichia	coli
		2.676	1.17E-08	Proteobacteria	Gammaproteobacteria	Enterobacteriales	Enterobacteriaceae	Serratia	entomophila
		2.888	0.0004	Proteobacteria	Gammaproteobacteria	Enterobacteriales	Enterobacteriaceae	Enterobacter	aceae
		3.011	0.007	Proteobacteria	Gammaproteobacteria	Enterobacteriales	Enterobacteriaceae	Enterobacter	
		3.22	1.03E-11	Proteobacteria	Gammaproteobacteria	Enterobacteriales	Enterobacteriaceae		
		3.608	4.49E-05	Firmicutes	Clostridia	Clostridiales	Veillonellaceae	Mitsuokella	
		4.005	0.0034	Proteobacteria	Gammaproteobacteria	Enterobacteriales	Enterobacteriaceae	Trabulsiella	guamensis
		4.611	0.0021	Proteobacteria	Gammaproteobacteria	Enterobacteriales	Enterobacteriaceae	Enterobacter	cowanii
		4.896	0.0011	Proteobacteria	Gammaproteobacteria	Enterobacteriales	Enterobacteriaceae	Yersinia	
		5.158	1.12E-06	Bacteroidetes	Bacteroidia	Bacteroidales	Prevotellaceae	Prevotella	multisaccharivorax
