# Supplementary material for: Household triclosan and triclocarban effects on the infant and maternal microbiome
Source: EMBO Mol Med. 2017 Oct 13;9(12):1732–41. doi: 10.15252/emmm.201707882 (PMC5709730; doi:10.15252/emmm.201707882)
Supplement: Supplementary file 7 — Source Data for Figure 4 [file EMMM-9-1732-s006.zip › Source_Data_figure4/EMM-2017-07882-SourceDataForFigure4BC.pdf]

Group	Visit (Months)	Enrichment	Log2 Fold Change	FDR adjusted p-value	Phylum	Class	Order	Family	Genus	Species
**Infant**	**2**	**nTC**	2.304	0.0235	Bacteroidetes	Bacteroidia	Bacteroidales	Bacteroidaceae	Bacteroides	xylanisolvens
			3.794	0.0196	Proteobacteria	Gammaproteobacteria	Enterobacteriales	Enterobacteriaceae	Citrobacter	freundii
			5.141	0.0196	Firmicutes	Clostridia	Clostridiales	Clostridiaceae	Clostridium	neonatale
			5.358	0.0196	Firmicutes	Erysipelotrichi	Erysipelotrichales	Erysipelotrichaceae	Eubacterium	dolichum
		**TC**	2.853	0.0241	Firmicutes	Clostridia	Clostridiales	Clostridiaceae	Clostridium	cavendishii
			3.232	0.0003	Bacteroidetes	Bacteroidia	Bacteroidales	Bacteroidaceae	Bacteroides	dorei
			3.241	0.0092	Proteobacteria	Gammaproteobacteria	Pasteurellales	Pasteurellaceae	Mannheimia	caviae
			5.5	0.0003	Firmicutes	Clostridia	Coriobacteriales	Coriobacteriaceae	Collinsella	aerofaciens
			5.53	0.0196	Firmicutes	Clostridia	Clostridiales	Clostridiaceae	Clostridium	perfringens
			5.626	1.78E-09	Actinobacteria	Actinobacteria	Bifidobacteriales	Bifidobacteriaceae	Bifidobacterium	dentium
**Infant**	**6**	**nTC**	5.07	0.0094	Bacteroidetes	Bacteroidia	Bacteroidales	Bacteroidaceae	Bacteroides	fragilis
			5.305	3.73E-05	Bacteroidetes	Bacteroidia	Bacteroidales	Porphyromonadaceae	Parabacteroides	merdae
		**TC**	1.766	0.0279	Firmicutes	Bacilli	Lactobacillales			
			4.048	0.0405	Proteobacteria	Betaproteobacteria	Burkholderiales	Alcaligenaceae	Sutterella	stercoricanis
			4.728	0.0005	Firmicutes	Clostridia	Clostridiales	Veillonellaceae	Veillonella	ratti
**Infant**	**10**	**nTC**	2	0.0068	Firmicutes	Clostridia	Clostridiales	Clostridiaceae	Clostridium	
			2.049	0.0139	Firmicutes	Clostridia	Clostridiales	Clostridiaceae	Clostridium	cavendishii
			2.295	0.0268	Firmicutes	Clostridia	Clostridiales	Clostridiaceae	Clostridium	taeniosporum
			2.982	0.035	Proteobacteria	Betaproteobacteria				
			3.036	0.0481	Firmicutes	Bacilli	Lactobacillales	Lactobacillaceae	Lactobacillus	siliginis
		**TC**	3.028	0.0025	Firmicutes	Bacilli	Lactobacillales	Streptococcaceae	Streptococcus	bovis
			4.037	0.0007	Bacteroidetes	Bacteroidia	Bacteroidales	Prevotellaceae	Prevotella	copri
			4.073	0.043	Verrucomicrobia	Verrucomicrobiae	Verrucomicrobiales	Verrucomicrobiaceae	Luteolibacter	algae
			4.753	0.035	Firmicutes	Bacilli	Lactobacillales	Streptococcaceae	Streptococcus	luteciae
			4.979	0.0095	Firmicutes	Bacilli	Lactobacillales	Streptococcaceae	Streptococcus	infantarius
			5.9	1.02E-07	Verrucomicrobia	Verrucomicrobiae	Verrucomicrobiales	Verrucomicrobiaceae	Akkermansia	muciniphila
			6.42	2.05E-07	Bacteroidetes	Bacteroidia	Bacteroidales	Bacteroidaceae	Bacteroides	caccae
**Mother**	**6**	**nTC**	1.377	0.0172	Firmicutes	Clostridia	Clostridiales	Lachnospiraceae	Lachnospira	pectinoschiza
			2.214	0.0138	Bacteroidetes	Bacteroidia	Bacteroidales	Bacteroidaceae	Bacteroides	sartorii
			2.474	0.0254	Firmicutes	Clostridia	Clostridiales	Lachnospiraceae	Lachnospira	
			2.516	0.0158	Bacteroidetes	Bacteroidia	Bacteroidales	Porphyromonadaceae	Dysgonomonas	wimpennyi
			3.791	0.0073	Bacteroidetes	Bacteroidia	Bacteroidales	Bacteroidaceae	Bacteroides	stercoris
			4.886	0.0158	Bacteroidetes	Bacteroidia	Bacteroidales	Bacteroidaceae	Bacteroides	intestinalis
			7.136	4.97E-05	Bacteroidetes	Bacteroidia	Bacteroidales	Bacteroidaceae	Bacteroides	massiliensis

	**TC**	1.568	0.0322	Firmicutes	Bacilli	Lactobacillales	Enterococcaceae	Enterococcus	
		1.78	0.0322	Proteobacteria	Gammaproteobacteria	Enterobacteriales	Enterobacteriaceae	Serratia	entomophila
		3.354	0.0322	Proteobacteria	Gammaproteobacteria	Enterobacteriales	Enterobacteriaceae	Trabulsiella	odontotermitis
		3.895	0.0073	Proteobacteria	Gammaproteobacteria	Aeromonadales	Aeromonadaceae	Tolumonas	auensis
		4.058	2.57E-07	Proteobacteria	Gammaproteobacteria	Enterobacteriales	Enterobacteriaceae		
		4.184	2.28E-06	Proteobacteria	Gammaproteobacteria	Enterobacteriales	Enterobacteriaceae	Enterobacter	amnigenus
		4.537	0.0158	Proteobacteria	Gammaproteobacteria	Enterobacteriales	Enterobacteriaceae	Erwinia	tasmaniensis
		5.266	0.028	Proteobacteria	Gammaproteobacteria	Enterobacteriales	Enterobacteriaceae	Trabulsiella	
		5.476	8.77E-07	Proteobacteria	Gammaproteobacteria	Enterobacteriales	Enterobacteriaceae	Citrobacter	werkmanii
		5.674	0.0119	Proteobacteria	Gammaproteobacteria	Enterobacteriales	Enterobacteriaceae	Citrobacter	
		6.703	0.0011	Proteobacteria	Gammaproteobacteria	Enterobacteriales	Enterobacteriaceae	Citrobacter	freundii
		7.584	0.0012	Proteobacteria	Gammaproteobacteria	Enterobacteriales	Enterobacteriaceae	Enterobacter	hormaechei
		7.923	6.66E-05	Proteobacteria	Gammaproteobacteria	Enterobacteriales	Enterobacteriaceae	Klebsiella	
**10**	**nTC**	3.302	0.0178	Bacteroidetes	Bacteroidia	Bacteroidales	Bacteroidaceae	Bacteroides	stercoris
		4.007	0.0237	Cyanobacteria	Nostocophycideae	Stigonematales	Rivulariaceae	Calothrix	parietina
	**TC**	1.322	0.0466	Bacteroidetes	Bacteroidia	Bacteroidales	Bacteroidaceae	Bacteroides	graminisolvens
		1.765	0.0012	Proteobacteria	Gammaproteobacteria	Enterobacteriales	Enterobacteriaceae		
		2.009	0.0012	Proteobacteria	Gammaproteobacteria	Enterobacteriales	Enterobacteriaceae	Serratia	entomophila
		2.464	0.0498	Proteobacteria	Gammaproteobacteria	Enterobacteriales	Enterobacteriaceae	Escherichia	albertii
		2.647	0.0466	Proteobacteria	Gammaproteobacteria	Enterobacteriales	Enterobacteriaceae	Serratia	
		2.706	0.0012	Firmicutes	Clostridia	Clostridiales	Lachnospiraceae	Oribacterium	sinus
		2.981	0.0034	Proteobacteria	Betaproteobacteria	Burkholderiales	Alcaligenaceae	Sutterella	
		3.038	0.0012	Proteobacteria	Betaproteobacteria	Burkholderiales			
		3.133	0.0012	Proteobacteria	Gammaproteobacteria	Enterobacteriales	Enterobacteriaceae	Escherichia	
		3.254	0.0059	Proteobacteria	Gammaproteobacteria	Enterobacteriales	Enterobacteriaceae	Escherichia	coli
		3.641	0.0374	Proteobacteria	Betaproteobacteria	Burkholderiales	Burkholderiaceae	Burkholderia	phenoliruptrix
